# Naturalistic study of the joint presence of headache and pets

**DOI:** 10.1186/1129-2377-14-S1-P35

**Published:** 2013-02-21

**Authors:** D Moscato, B Calabrese, FR Moscato

**Affiliations:** 1 IDI Sanità, via Monti di Creta 104 00165 Roma, Italy

## Introduction

Pet Therapy is our first choice intervention for the therapy of children’s headache, since in the majority of children in growing up age headache is often linked to a situation of psycho-social discomfort [1]. On the basis of several works, which had found that just the simple presence of pets was an improvement factor of the physical conditions of several patients [2,3], we wanted to ascertain whether also the simple presence of pets (mammals) could be related to the development of childhood headache.

## Methods

In a sample chosen in compulsory schools of our district we administered a questionnaire that would use (IHS, 2004) for the diagnosis of headache in the fifth year of primary school. The questionnaire, in addition to the data relating to the number of brothers and sisters and social conditions, indicated the presence of pets (mammals) in the family nucleus. results 477 children participated in the study (279f.198m. range 10-12 years), with diagnosis of Migraine 10.3%(8.4%MwA, 1.9MWA )Tension Type Headache17.4%(FTTH14 %, CTTH 3.5%). No significant differences were found in the number of brothers and sisters, and in the social conditions. The presence of pets was equal to 18.4% of healthy children, whilst it was 4.3% in migraine sufferers, compared to 4.8% in children suffering from tension type headache.

## Conclusion

The presence of animals in the house is significantly concurrent with a lower incidence of migraine and tension headache. The presence of pets in the house seems to be a factor of prevention of the onset of headache. From an epidemiologic standpoint, the interaction with a pet presupposes a difference of family lifestyle and a consequent development in the coping modality, enabling to mitigate the arising of those etiological cognitive factors, which can promote headache suffering.
